# Comparative genomic analysis and expression of the *APETALA2*-like genes from barley, wheat, and barley-wheat amphiploids

**DOI:** 10.1186/1471-2229-9-66

**Published:** 2009-05-29

**Authors:** Javier Gil-Humanes, Fernando Pistón, Antonio Martín, Francisco Barro

**Affiliations:** 1 Departamento de Mejora Genética Vegetal. Instituto de Agricultura Sostenible, CSIC, 14080-Córdoba, Spain

## Abstract

**Background:**

The *APETALA2*-like genes form a large multi-gene family of transcription factors which play an important role during the plant life cycle, being key regulators of many developmental processes. Many studies in *Arabidopsis *have revealed that the *APETALA2 *(*AP2*) gene is implicated in the establishment of floral meristem and floral organ identity as well as temporal and spatial regulation of flower homeotic gene expression.

**Results:**

In this work, we have cloned and characterised the *AP2*-like gene from accessions of *Hordeum chilense *and *Hordeum vulgare*, wild and domesticated barley, respectively, and compared with other *AP2 *homoeologous genes, including the Q gene in wheat. The *Hordeum AP2*-like genes contain two plant-specific DNA binding motifs called AP2 domains, as does the Q gene of wheat. We confirm that the *H. chilense AP2*-like gene is located on chromosome 5H^ch^. Patterns of expression of the *AP2*-like genes were examined in floral organs and other tissues in barley, wheat and in tritordeum amphiploids (barley × wheat hybrids). In tritordeum amphiploids, the level of transcription of the barley *AP2*-like gene was lower than in its barley parental and the chromosome substitutions 1D/1H^ch ^and 2D/2H^ch ^were seen to modify *AP2 *gene expression levels.

**Conclusion:**

The results are of interest in order to understand the role of the *AP2*-like gene in the spike morphology of barley and wheat, and to understand the regulation of this gene in the amphiploids obtained from barley-wheat crossing. This information may have application in cereal breeding programs to up- or down-regulate the expression of *AP2*-like genes in order to modify spike characteristics and to obtain free-threshing plants.

## Background

One of the main objectives of cereal breeding is to expand genetic variability within cultivated species. Wild species, related to cultivated crops, are an important source of variability. Inter-specific hybridization can be used to introgress genetic variability from wild species into crops and to produce new species with valuable agronomic traits. An example of this is the hexaploid tritordeum, an amphiploid obtained by crossing *Triticum turgidum *L. (Thell) (2n = 4x = 28) with *Hordeum chilense *(Roem. et Schult.) (H^ch^H^ch^, 2n = 2x = 14). Primary tritordeums exhibit enormous genetic variability for many valuable agronomic and quality traits. For example, the grain and flour from tritordeum has similar functional properties to bread wheat [1], but with higher pigment content [2,3]. Most of this genetic variability can be attributed to *H. chilense*, a wild relative of cultivated barley (*H. vulgare *L.) that occurs exclusively in Chile and Argentina *which *is highly polymorphic both morphologically and biochemically. The variability for important agronomic traits, such as endosperm storage proteins [4,5], carotenoid content [6] and resistance to biotic stresses [7], linked to its high crossability, makes *H. chilense *a suitable candidate as a source of genetic variability for the transfer of useful genes to wheat by wide crossing. However, in the process of hybridization, undesirable traits such as rachis brittleness and non-free threshing characters, present in wild barley, are also transferred to the hybrid, limiting its use as an alternative cereal crop.

Many genetic systems have been proposed as responsible for the free-threshing character in hexaploid wheat. MacKey [8] proposed a polygenic system distributed throughout the three genomes that counteracts glume tenacity and rachis brittleness. A second system is related to the major gene or gene complex Q [9,10] located in the long arm of the chromosome 5A which governs the free-threshing character and square spike phenotype. In addition, the Q gene pleiotropically influences many other characters determinant for domestication such as rachis fragility [9,11], glume shape and tenacity [10,12], spike length [10,13], plant height [10,13,14] and spike emergence time [13]. Other genes which influence the free-threshing habit include the *Tg *locus, located on chromosome 2D [11,15] that codes for tenacious glumes and is thought to inhibit the expression of the Q gene.

Simons *et al*. [16] have cloned and characterized the Q gene in wheat and showed that it has a high homology to members of the APETALA2 (AP2) family of transcription factors. This gene family is characterized by two plant-specific DNA binding motifs referred to as AP2 domains. The *AP2 *genes form a large multigene family, and play multiple roles during the plant life cycle being key regulators of many developmental processes such as floral organ identity determination or control of leaf epidermal cell identity [17]. Many studies in *Arabidopsis *have revealed that the *AP2 *gene is implicated in the establishment of floral meristem identity [18,19], floral organ identity [20-22] and the temporal and spatial regulation of flower homeotic gene expression [23].

In the present work, we have cloned and characterised an *AP2*-like gene from accessions of *H. chilense *and *H. vulgare*, wild and domesticated barley respectively, and compared these with other homoeologous genes, including the Q gene from wheat. The pattern of expression of the *AP2*-like gene in floral organs and other tissues in barley, wheat and amphiploid tritordeum was also studied. The results are relevant to understanding the role of the *AP2*-like gene in the spike morphology of barley and wheat and in hybrids obtained from their crossing and for modification of the expression of *AP2*-like genes to modify the spike characteristics of cereals for breeding purposes. In addition, the results provide insight into how important agronomic genes such as *AP2 *are regulated in cereal hybrids.

## Results

### Structure of the *AP2*-like genes from *H. vulgare *and *H. chilense *and their predicted proteins

**Figure 1:**
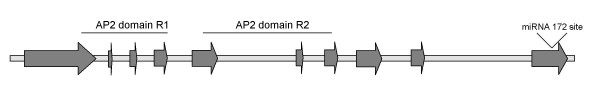
**Illustrated structure of the *AP2*-like gene in wild (*H. chilense*) and cultivated (*H. vulgare*) barley**. Exons are represented by arrows and introns by grey bars. AP2 domains and the *miRNA172 *binding site are also represented.

Genomic DNA and complete cDNA sequences obtained in this work from *H. vulgare *cv Betzes (line H106) and *H. chilense *(lines H1 and H208), and their predicted proteins were searched using BLASTn and BLASTp algorithms. Results showed a high homology to many floral homeotic genes and their corresponding proteins such as the *T. aestivum *floral homeotic (*Q*) mRNA [GenBank: AY702956, AAU94922], the *H. vulgare AP2*-like mRNA [GenBank: AY069953, AAL50205], the *Zea mays *indeterminate spikelet 1 (*ids1*) mRNA [GenBank: AF048900, AAC05206], the *Oryza sativa *transcription factor *AP2D2 *mRNA [GenBank: AY685113, AAO65862] and the *A. thaliana APETALA2 *[GenBank: U12546, AAC13770]. All these genes belong to an AP2 subfamily of putative transcription factors which are characterized by the presence of two DNA binding motifs, referred to as AP2 domains, which consist of 60 and 61 conserved amino acids, respectively (Figure 1). Predicted proteins for *H. vulgare *cv Betzes and *H. chilense *lines reported here, also revealed the presence of these AP2 domains in the deduced amino acid sequences. The structure of the *AP2*-like genes of the *Hordeum *genotypes is similar to that of other *AP2*-like genes (Figure 1). They all presented 10 exons and 9 introns, and the 21 nt microRNA binding site (miRNA172), which is highly conserved in all *AP2*-like genes with only a single nucleotide change in the *T. aestivum *cv Chinese spring AP2-like sequence [GenBank: AY702956].

Table 1 summarizes the characteristics of the genomic DNA, its open reading frame (ORF) and the resulting protein of the *AP2*-like genes from *H. vulgare *cv. Betzes (H106) and *H. chilense *lines H11 and H208, and comparisons with *H. vulgare *cv. Forester and *T. aestivum *cv Chinese spring. In genomic DNA gene length ranged from 3229 bp in *T. aestivum *to 3244 bp in *H. chilense *line H11 while the ORF extended 1323 bp in all the genotypes except in *T. aestivum*, which was slightly longer with 1344 bp. Therefore, the protein length was 447 amino acids for *T. aestivum *and 440 amino acids for the rest of genotypes. The GC content was higher for all genotypes in the ORF (64.1% to 65%) than in genomic DNA (51.4% to 53.3%). The estimated molecular weight of the proteins was around 48–49 KDa while their theoretical isoelectric points (pI) were between 6.72 and 7.31. The percentage of identity and polymorphism of the sequences were estimated by means of a comparative alignment of all the genotypes with the *T. aestivum *cv Chinese Spring [GenBank: AY702956]. The percentage of identity ranged from 82.1% to 83% in the genomic DNA, from 91.9% to 92.6% in the ORF and from 89.8% to 91.8% at protein level (Table 1). These data confirm the high resemblance between the genotypes at transcript and protein levels.

**Table 1:** Description of the *AP2*-like genes (genomic DNA, ORF and predicted protein) of *H. vulgare *cv. Betzes (H106), *H. vulgare *cv. Forester, *H. chilense *lines H11 and H208 and *T. aestivum *cv Chinese spring.

	**Genomic DNA^(1)^**				**ORF**		**Protein**			**% of identity^(2)^**		
				
	Length (bp)	GC%	Number of		Length (bp)	GC%	Length (aa)	MW (KDa)	pI	Genomic DNA	ORF	Protein
			Introns	Exons								
***T. aestivum***												
**cv. Chinese spring** [GenBank: AY702956]	3229	53.3	9	10	1344	65	447	48.968	6.72	100	100	100
***H. vulgare***												
**cv. Betzes (H106)**	3234	51.4	9	10	1323	64.1	440	48.424	7	82.1	92	91.5
**cv. Forester **[GenBank: AY069953]	n/a	n/a	n/a	n/a	1323	64.2	440	48.081	7.31	n/a	91.9	89.8
***H. chilense***												
**H11**	3244	52.2	9	10	1323	64.3	440	48.315	6.94	82.9	92.5	91.3
**H208**	3240	52.3	9	10	1323	64.6	440	48.273	6.97	83	92.6	91.8

^(1) ^DNA from start to end codons

^(2) ^The percentage of identity was estimated by a comparative alignment of all sequences with that of *H. vulgare *cv. Betzes (H106)

The ORFs from *H. vulgare *cv Betzes (line H106) and *H. chilense *(lines H11 and H208) *AP2*-like genes were aligned and compared. Table 2 shows all the nucleotide changes (single nucleotide polymorphisms (SNP), insertions and deletions) and their positions with respect to *H. vulgare *cv Betzes (line H106). Fifty-two polymorphisms were observed among the three genotypes, three of them were insertions/deletions (Ins/Del) and the rest were SNPs. Only sixteen of the polymorphisms (indicated in bold and asterisk) caused changes in the amino acid sequence.

**Table 2:** Nucleotide polymorphism analysis of the *AP2 *open reading frame (ORF) in *H. vulgare *line H106 and *H. chilense *lines H11 and H208.

	**Genotype**				**Genotype**		
			
**position**	**H106**	**H11**	**H208**	**position**	**H106**	**H11**	**H208**
21	C	G	G	618	G	C	C
27	T	G	G	708	G	T	T
33	C	G	G	711	C	T	T
63	C	T	T	738	G	C	C
80*	CCG	-	-	744	C	T	T
86	C	G	G	754*	A	C	C
120	C	T	T	768	G	A	A
137*	A	G	G	771	A	G	G
144	G	T	T	774*	T	A	A
180	G	C	C	817*	A	C	C
190*	C	T	T	861	T	T	G
210	C	G	G	874	A	C	C
219	G	C	C	914*	A	G	G
233*	C	T	C	954	G	A	A
258	A	G	G	975	C	T	T
261	T	G	G	1086	C	G	G
300	C	G	G	1117*	T	A	A
304*	A	G	G	1139*	C	A	A
305*	A	C	C	1141*	T	C	C
354	C	G	C	1143*	C	G	G
402	T	C	C	1212	T	C	C
450	C	T	T	1270*	-	GCGCCG	GCGCCG
453	A	G	G	1285*	TCA	-	-
576	T	C	C	1289*	C	T	C
597	C	G	G	1294	C	G	G
612	G	A	A	1315	G	C	C

All the nucleotide changes (SNPs, insertions and deletions) and their positions with respect to the sequence of *H. vulgare *cv Betzes (line H106) are shown. Polymorphisms causing changes in the amino acid sequence are indicated in bold and with asterisks.

**Figure 2:**
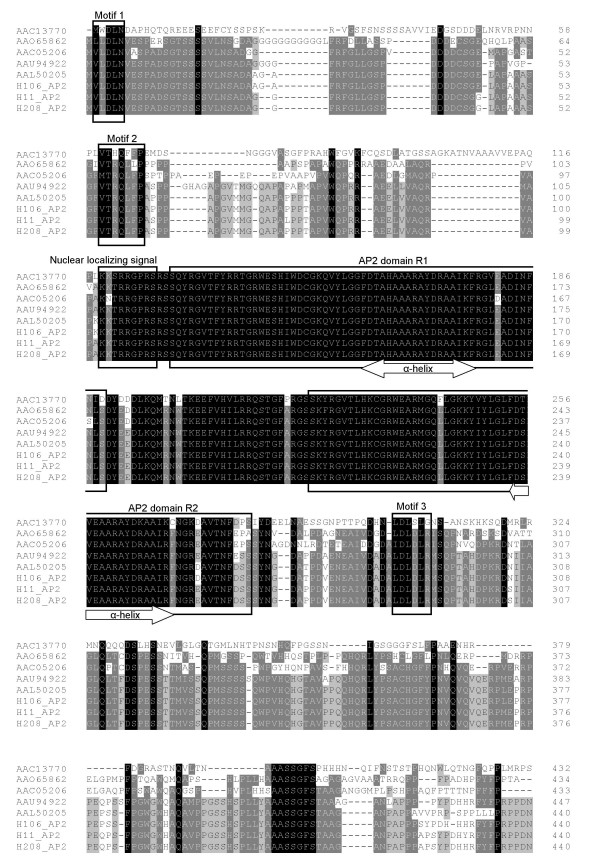
**Alignment of the AP2-like proteins of *Arabidopsis thaliana *(AAC13770), *Oryza sativa ***[GenBank: AAO65862]**, *Zea mays ***[GenBank: AAC05206]**, *Triticum aestivum ***[GenBank: AAU94922]**, *Hordeum vulgare *cv. Forester **[GenBank: AAL50205]** and the predicted protein from the *AP2*-like gene of *H. vulgare *cv. Betzes (H106) and *H. chilense *lines H11 and H208**. Different features (motif 1, motif 2, nuclear localizing signal, AP2 domains R1 and R2, and motif 3) are boxed. The α-helical structures located in the core region of each AP2 domain are delimited by arrows.

Comparison of the AP2 predicted proteins from *H. vulgare *cv. Betzes (H106) and *H. chilense *lines H11 and H208, with other AP2-like proteins is showed in Figure 2. All the AP2-like proteins compared had similar structural organizations: Motif 1, Motif 2, the nuclear localizing signal, the first AP2-domain (AP2 R1), the second AP2-domain (AP2 R2), and Motif 3. The two AP2 domains were strongly conserved among the different species. Hence, the two AP2 domains were almost identical in the three Triticeae species compared (*T. aestivum, H. chilense *and *H. vulgare*) with only a single amino acid change in the *H. vulgare *sequences (position 258 of the protein) and another in the *T. aestivum *sequence (position 269). The three motifs and the nuclear localizing signal were also highly conserved in all the species aligned in Figure 2. The relationships among the AP2-like proteins was confirmed by constructing a phylogenetic tree based on the the neighbor-joining method (Additional File 1: Phylogenetic tree of the AP2-like proteins). The resulting tree showed that the *Hordeum *genotypes clustered together and very close to *T. aestivum *while the rest of the genotypes were distant.

### Chromosomal location of the *AP2*-like gene in *H. chilense*

**Figure 3:**
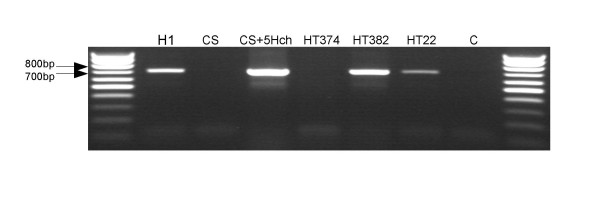
PCR of the barley *AP2 *genomic sequence using the primers pair AP2*F2/AP2Hch*R2 with different genotypes of *H. chilense *(H1), *T. aestivum *(cv. Chinese Spring and a 5H^ch ^addition line of cv. Chinese spring), and tritordeum (HT374, HT382 and HT22).

The chromosomal location of the *AP2*-like gene in *H. chilense *was demonstrated by using a 5H^ch ^addition line of wheat, a 5D/5H^ch ^substitution line of tritordeum (HT374), and a 1D/1H^ch ^and 2D/2H^ch ^double substitution line of tritordeum (HT382). Figure 3 shows the result of the amplification by PCR of a sequence of the genomic barley *AP2-*like gene using the pair of primers AP2*F2/AP2Hch*R2 and the DNA isolated from the above genotypes. Amplification of the barley *AP2 *was obtained in genotypes carrying the 5H^ch ^chromosome, these are *H. chilense *H1, the 5H^ch ^addition line of *T. aestivum *cv. Chinese Spring, and the tritordeum lines HT382 (1D/1H^ch^, 2D/2H^ch^) and HT22. In contrast, the wheat cv. Chinese spring and the tritordeum line HT374 (5D/5H^ch^) did not show amplification (Figure 3). This result shows that the *AP2*-like gene in *H. chilense *is located in the chromosome 5H^ch ^and tritordeum line HT374 (5D/5H^ch^) lacks the 5H^ch ^chromosome and therefore the *AP2*-like gene.

### Quantitative real-time PCR of *AP2*-like genes

**Figure 4:**
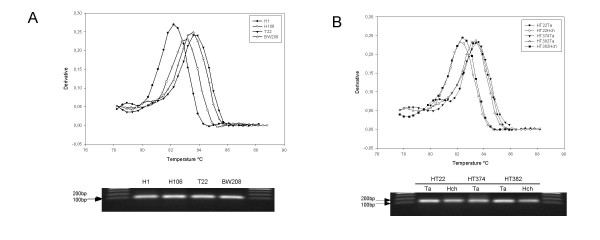
**Dissociation curves and agarose gel electrophoresis of the wheat *AP2 *and barley *AP2 *amplification products**. (A) Dissociation curves and agarose gel electrophoresis of the *AP2 *qRT-PCR products of genotypes H1 (*H. chilense*), H106 (*H. vulgare *cv Betzes), T22 (*T. durum*) and BW208 (*T. aestivum*). (B) Dissociation curves and agarose gel electrophoresis of the wheat *AP2 *(Ta) and barley *AP2 *(Hch) specific qRT-PCR products of the three tritordeum lines HT22, HT374 and HT382.

The expression level of the *AP2*-like genes were determined in different tissues by qRT-PCR in wild (H1) and domesticated (H106) barley, durum wheat (T22), bread wheat (cv Bobwhite 'BW208'), and tritordeum (HT22, HT374 and HT382). We designed two sets of primers to specifically amplify a fragment of the *AP2*-like cDNA corresponding to wheat genome (AP2*F2/AP2Ta*R2) and *Hordeum *genome (AP2*F2/AP2Hch*R2) respectively (Table 3). Therefore, primer pair AP2*F2/AP2Ta*R2 was used to quantify the expression of the wheat *AP2-*like gene in durum and bread wheat (genotypes T22 and BW208) as well as in tritordeum genotypes HT22, HT374 and HT382. On the other hand, AP2*F2/AP2Hch*R2 primers were used to quantify the expression barley *AP2*-like gene in *H. chilense *(H1), *H. vulgare *(H106), and in the above tritordeum genotypes. The amplification result of each pair of primers is shown in Figure 4. The qRT-PCR product of each reaction had a unique melting temperature peak, indicating that specific amplification occurred. Figure 4A shows the dissociation curves and agarose gel electrophoresis of genotypes H1, H106, T22 and BW208. The two peaks corresponding to *Triticum *genotypes (T22 and BW208) had the same melting temperature (83.6°C), while the wild barley (H1) peak had a lower melting temperature (82.2°C). The cultivated barley dissociation curve (H106) presented a melting temperature of 83.2°C. The expected product size was 104 bp for *Triticum *genotypes and 108 bp for both wild and cultivated barley. This fragment of 108 bp contains 8 SNP differences between wild (H11 and H208) and cultivated barley (H106) and is identical in H11 and H208. Consequently, differences in melting temperature of PCR products between wild and cultivated barley can be explained by those 8 SNPs found between the two sequences. Figure 4B shows the dissociation curves and agarose gel electrophoresis of the specific amplification of the wheat and barley *AP2*-like genes using the three tritordeum lines HT22, HT374 and HT382. As above, the predicted product size was 104 bp and 108 bp for wheat and barley *AP2*-like genes, respectively. Although this 4 nucleotide difference could not be observed in the agarose gel, different melting temperatures were detected for each PCR product, indicating that specific amplification occurred in tritordeum background. In addition, melting temperatures of PCR products in tritordeum lines of both wheat *AP2*-like gene and barley *AP2*-like gene were similar to that in wheat genotypes and wild barley respectively, as described above (Figure 4B).

**Table 3:** PCR primers used in this work

**Primer**	**Description**	**Sequence (5'-3')**
**F1APT2**	external forward for 3' RACE	CTGGAGGCCGACATCAACTTCAATCTG
**F2APT2**	nested forward for 3' RACE	GGAGCTCSAAGTACCGCGGCGTCAC
**R1APT2**	external reverse for 5' RACE	TGGGTACAAACGCTGGTGCTGCTGAG
**R2APT2**	nested reverse for 5' RACE	AAGGCCGGCGATGATGTTGTCCCTCTTG
**AP2F4**	forward for "edge-to-edge"	CGGCCACCGCGCTCCCATGCCATA
**AP2 new R**	reverse for "edge-to-edge"	CACACCCGTCGACCRCCGTCCAT
**F3APT2**	forward for DNA sequencing	GAYTGCGGGAAGCAGGTCTACTTG
**F4APT2**	forward for DNA sequencing	AGATGCTMCACCTGACGTCGAAAATGAG
**R5APT2**	reverse for DNA sequencing	CTTCAACTTCGCTGTCRAAMAGCCCAAGAT
AP2*F2	forward for qRT-PCR	GGCCGCTGGGAGGCAAGGATGG
AP2Ta*R2	reverse for qRT-PCR (genomes A, B and D)	GCCGCCCTGTCGTACGCCCTTG
AP2Hch*R2	reverse for qRT-PCR (genome H)	GAAGCGCCGCCCTATCGTAGGCTCT
**Actin F4**	forward for actin gene	ACCTTCAGTTGCCCAGCAAT
**Actin R4**	reverse for actin gene	CAGAGTCGAGCACAATACCAGTTG

**Figure 5:**
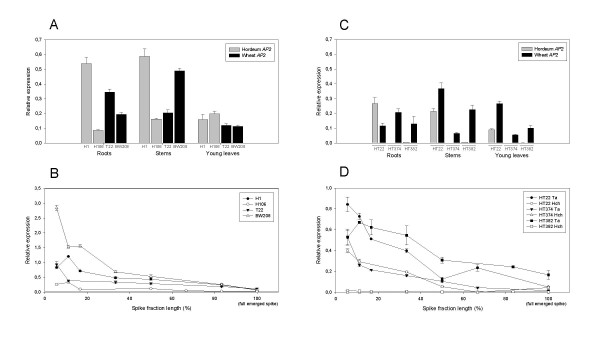
**Relative expression of the *AP2*-like gene in roots, stems, leaves and developing spikes in different genotypes**. (A) and (B) Relative expression in genotypes H1 (*H. chilense*), H106 (*H. vulgare *cv Betzes), T22 (*T. durum*) and BW208 (*T. aestivum*). (C) and (D) Relative expression of the wheat *AP2 *and barley *AP2*-like genes in three tritordeum lines (HT22, HT374 and HT382).

**Figure 6:**
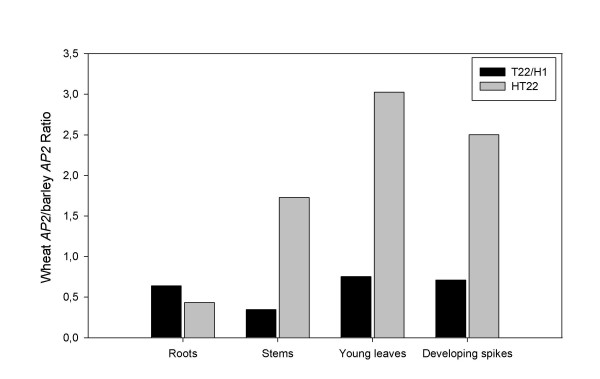
**Ratio between the expression of wheat *AP2 *and barley *AP2 *(wheat *AP2*/barley *AP2*) in different tissues of the tritordeum line HT22 and its parents, H1 (*H. chilense*) and T22 (*T. durum*)**.

The expression of wheat and barley *AP2*-like genes was determined in roots, stems, young leaves, and spikes at various developmental stages, and normalized with the expression of the *actin *gene as a reference. Figure 5A and 5B compare the relative expression of the *AP2*-like gene in wild (H1) and cultivated (H106) barley, and in durum (T22) and bread (BW208) wheat. Expression of the *AP2*-like gene was detected in all the tissues and genotypes. In roots and stems, wild barley (H1) had higher transcription levels than cultivated barley (H106). In turn, durum wheat had higher expression levels in roots but lower in stems than that of bread wheat. All four genotypes showed similar expression levels in young leaves. In the case of spikes, expression levels of the *AP2*-like gene decreased in all genotypes over the course of development and emergence of the spike (Figure 5B). Figures 5C and 5D compare the level of transcription of the corresponding wheat and barley *AP2*-like genes in the three tritordeum lines tested. Line HT374 has the 5D/5H^ch ^substitution whereas line HT382 has the double substitution 1D/1H^ch ^and 2D/2H^ch^. Finally, line HT22 is a tritordeum amphiploid with no chromosome substitution. Line HT22 showed higher expression of the barley *AP2*-like gene in roots but lower than that of wheat in stems and young leaves. Line HT374 lacks the 5H^ch ^chromosome and therefore the expression of the barley *AP2*-like gene was not detected in this genotype in roots, stems and young leaves (Figure 5C). Although line HT382 contains the 5H^ch ^chromosome, and therefore the barley *AP2*-like gene (Figure 3), its expression was strongly reduced in roots, stems and young leaves (Figure 5C). Finally, in developing spikes, the expression levels of the wheat *AP2*-like gene in tritordeum HT22 was higher than that of barley (Figure 5D). In line HT374 the expression of the AP2-like gene was not detected whereas in line HT382 its remains at a low level during spike development (Figure 5D),

The ratio between the expression levels of wheat and barley *AP2-*like genes (wheat *AP2*/barley *AP2*) in different tissues of the amphiploid tritordeum HT22 was calculated and compared with transcription levels in the corresponding H1 (*H. chilense*) and T22 (*T. durum*) parentals (Figure 6). In all the tissues studied, the level of transcription of the wheat *AP2*-like gene in durum wheat (T22) was lower than the transcription level of the barley *AP2*-like gene in wild barley (H1) and therefore, the wheat/barley AP2 ratio was below 1. In contrast, in the amphiploid tritordeum (HT22), the wheat *AP2-*like gene was transcribed at higher levels in all tissues, except in roots. Consequently the wheat/barley AP2 ratio was higher in the amphiploid HT22 than in its corresponding H1 and T22 parentals. The wheat/barley AP2 ratio in the amphiploid was higher in young leaves and developing spikes than that of roots and stems (Figure 6).

## Discussion

Alignments of the sequenced cDNA and DNA of the *H. chilense *lines H11 and H208, and *H. vulgare *line H106 have shown that the internal structure of exons and introns of the *AP2*-like gene described in this work (Figure 1) is the same as that reported for the Q gene in wheat [GenBank: AY702956] [16], with 10 exons and 9 introns. BLAST searches revealed that the sequenced genes belong to the AP2 family of transcription factors which includes the floral homeotic gene *AP2 *[22] and *AINTEGUMENTA *(*ANT*) [24,25] involved in lateral organ development by controlling cell number and growth [26,27]. *AP2*-like genes are distinguished by having two plant-specific DNA binding motifs called AP2 domains and function as key developmental regulators in reproductive and vegetative organs [17]. Additionally, the *AP2*-like genes possess one *microRNA172 *binding site in the 3' region (Figure 1). The microRNA miR172 with 21-nucleotide non-coding RNA was reported to down-regulate several *Arabidopsis *genes in the AP2 subfamily [28,29]. This miR172 and its target sequence are highly conserved in all the genotypes aligned in Figure 2, with only a single change in the sequence of the *T. aestivum *cv Chinese spring [GenBank: AY702956].

The conserved DNA binding motifs, referred to as AP2 domains R1 and R2, consist of 60 and 61 amino acids respectively and the alignment with other AP2-like proteins confirmed that these domains are highly conserved, even between non-related species such as *H. chilense *and *A. thaliana *(Figure 2). Jofuku *et al*. [22] isolated and characterized the *AP2 *gene from *A. thaliana *[GenBank: U12546] and reported a 53% of identity between the two AP2 domains R1 and R2 in the central core of the AP2 polypeptide. They also described the presence of an 18-amino acids conserved core region in the two AP2 domains with a 72.2% of identity between them. This region is theoretically capable of forming amphipathic α-helical structures that may participate in protein-protein interactions. This α-helical structure could also participate in DNA binding, perhaps through the interaction of its hydrophobic face with the major groove of the DNA, as described for other proteins with similar α-helical structure [30]. In the study reported here, the AP2 R1 domain from *H. chilense *line H11 has 95% of identity with the corresponding amino acid sequence of that from *A. thaliana *described by Jofuku *et al*.[22], while the conserved core region is identical. On the other hand, when comparing the AP2 R2 domain of the two species we found 83.6% of identity in the full domain and 72.2% in the core region. Jofuku *et al*. [22] also reported the presence of a highly basic 10-amino acids domain adjacent to the AP2 R1 domain that included a putative nuclear localization sequence KKSR [31,32] which suggested that the AP2 may function in the nucleus. The same domain of 10 amino acids is present in the sequences reported here with only a change in the second position of the nuclear localization sequence (threonine instead serine) which is conserved in all the *Triticeae *sequences of the AP2-like proteins aligned in Figure 2. Tang *et al*. [33] described the amino acid sequences of the three motifs found in the AP2-like protein in rice and compared it with other AP2-like proteins from different species. We have found similar structures in the predicted AP2-like proteins of wild (H11 and H208) and cultivated (H106) barley (Figure 2).

Previous experiments involving the cytogenetic analysis of aneuploids have located the Q gene in the long arm of chromosome 5A of the wheat genome [9,34]. The *H. chilense *genome has been demonstrated to be collinear to other *Triticeae *genomes including those of bread wheat and *H. vulgare *[35] so that the *AP2*-like gene was predicted to be on chromosome 5H^ch ^of *H. chilense*. To confirm this, the *AP2*-like gene from *H. chilense *was amplified by PCR in *H. chilense *(H1), *T. aestivum *cv. Chinese Spring, a 5H^ch ^addition line of *T. aestivum *cv. Chinese spring, an amphiploid tritordeum (HT22), and two tritordeum lines HT374 and HT382 carrying chromosome substitutions (Figure 3). The results confirmed that the barley *AP2*-like gene is located on chromosome 5H^ch ^and that the HT374 substitution line (5D/5H^ch^) effectively lacks this chromosome.

Expression of the *AP2*-like gene was detected in all tissues studied. The transcription level peaked in the early stages of spike development and gradually decreased with spike maturation, being very similar in all the genotypes tested when the spike was fully emerged. These results are similar to those reported by Simons *et al*. [16] who observed higher expression of the Q gene in developing spikes, with a peak at the first stages of spike growth in wheat genotypes. Simons *et al*. [16] also reported lower expression in non-floral organs such as leaves and roots. Jofuku *et al*. [22] studied the transcription of *AP2 *in *A. thaliana *obtaining expression in floral organs (sepals, petals, stamens, carpels, developing ovules and inflorescence meristems) and also in non-floral organs (stems and leaves).

We characterised the expression of the *AP2*-like gene in wild (*H. chilense *genotype H1) and cultivated barley (*H. vulgare *genotype H106) (Figure 4) and found a higher level of transcription in wild barley in roots, stems and developing spikes. Simons *et al*. [16] reported that the transcription level of the *Q *allele, contained in bread and durum wheat cultivars, was consistently higher than that of *q *allele, contained in wild wheats, and this was related with differences in spike morphology. They also described that the single amino acid difference found in their predicted proteins could provide higher efficiency in homodimer formation in the *Q *allele with respect to that of the q allele. They suggested that the Q protein homodimer complex recognizes a region on its own promoter, enhancing the expression of the *Q *allele and leading to higher levels of the Q protein, and this was related to phenotypic differences in spike morphology between cultivated and wild wheats. Despite this, differences between Q and q can be compensated by a gene dosage effect, with 2.5 doses of q being equal to 1 dose of Q [10].

Our results with wild and cultivated barley showed that *H. chilense *(wild) had higher *AP2*-like gene expression levels than *H. vulgare *(cultivated). According to the model proposed by Simons *et al*., [16], in the case of barley, the homodimer formation should be higher in *H. chilense *than that of *H. vulgare*, resulting in higher transcript levels of the *AP2*-like gene in *H. chilense*. As for wheat, differences in expression levels for the *AP2*-like gene in wild and cultivated barley could be responsible for phenotypic differences in spike morphology. However, in barley, the line showing higher expression levels of the *AP2*-like gene is *H. chilense *which is not the cultivated phenotype. Hence, the wheat model does not entirely fit to barley, either because the mechanism in barley is different or because the mechanism is more complex than that reported by Simons et al. [16]. In the case of wheat, the *Q *and *q *alleles differ only by one amino acid while the *AP2*-like genes from wild and cultivated barley differ by 17 amino acids. Therefore, differences in the spike morphology between wild and cultivated barley may not only be due to *AP2-*like gene expression level, but additional elements must be added to the model to explain better the contribution of the *AP2*-like gene to spike morphology in barley.

We have also characterised the expression of the *AP2*-like genes corresponding to wheat and barley genomes in tritordeum amphiploids (HT22, HT374 and HT382). The comparison of the expression as wheat/barley AP2 ratio when both genes are expressed together in the tritordeum amphiploid (HT22) and when they are expressed separately in their parental lines, *H. chilense *(H1) and *T. durum *(T22), showed that the relative expression of the wheat *AP2*-like gene in different tissues was very constant in the amphiploid and in its durum wheat parental (T22). However, the level of transcription of the barley *AP2*-like gene was 3.5 times lower in the amphiploid than in its barley parental (H1). This could be explained by the presence of a mechanism of regulation for the barley *AP2*-like gene that makes it differently expressed in the amphiploid and in barley. One of the consequences of this down-regulation of the barley *AP2*-like gene may be the aspect of the spike in the tritordeum amphiploid, which is more similar to that of durum wheat than that of wild barley. However, many important agronomic features of the spike in tritordeum, such as fragile rachis and non-free threshing habit are similar to *H. chilense*.

Two tritordeum substitution lines (HT374 and HT382) with free-threshing habit [36] showed only expression of the wheat *AP2*-like gene. This was predictable for line HT374, which has a 5D/5H^ch ^chromosome substitution and consequently lacks the barley *AP2*-like gene, but not for line HT382 (double substitution 1D/1H^ch ^and 2D/2H^ch^) which carries the 5H^ch ^chromosome and therefore the barley *AP2*-like gene. Thus in this case, expression of the barley *AP2*-like gene was affected also by substitution of chromosomes 1H^ch ^and/or 2H^ch^. Atienza *et al*. [36] proposed that the presence of a homoeologous q locus on chromosome 5H^ch ^would be responsible for the non-free-threshing habit in tritordeum, and consequently the substitution of 5D/5H^ch ^in the line HT374 was responsible of the free-threshing habit. It was also suggested that the absence of chromosome 2H^ch ^conferred the free-threshing habit in tritordeum HT382, because of the absence of a homoeologous *Tg *locus from *H. chilense *that codes for tenacious glumes, in spite of the presence of q H^ch ^locus on chromosome 5H^ch ^[36]. Kerber *et al*. [15] and Jantasuriyarat *et al*. [11] have proposed that the action of *Tg *during flower development directly or indirectly interferes with the Q gene. The results reported from our work support this hypothesis as it appears that the absence of chromosome 2H^ch ^in tritordeum HT382 affects on the expression of the barley *AP2*-like gene, reducing its transcription to low levels.

## Conclusion

The *AP2*-like gene from wild and cultivated barley has been characterised in this work. The *AP2*-like genes contain two plant-specific DNA binding motifs called AP2 domains as does the Q gene of wheat. The results confirmed that the barley *AP2*-like gene is located on chromosome 5H^ch^. The expression of the *AP2*-like genes were studied in wheat, barley and tritordeum amphiploids showing that the level of transcription of the barley *AP2*-like gene in tritordeum was lower than in its barley parental. The chromosome substitutions 1D/1H^ch ^and 2D/2H^ch ^influence the expression of the barley AP2 in tritordeum. The results are of interest in understanding the role of the *AP2*-like gene in the spike morphology of cereals and in understanding the regulation of this gene in barley × wheat amphiploids. In addition, this information may be used in breeding programs for regulating the expression of *AP2*-like genes to modify spike characteristics and to obtain free-threshing plants.

## Methods

### Plant material

Plants used in this study were from the germplasm collection of the Instituto de Agricultura Sostenible (CSIC, Cordoba, Spain), and included *H. chilense *accessions H1, H11 and H208 (2n = 2x = 14; H^ch^H^ch^), *H. vulgare *cv Betzes (H106) (2n = 2x = 14; HH), *Triticum durum *accession T22 (2n = 4x = 28; AABB), *T. aestivum *cv Bobwhite (2n = 6x = 42; AABBDD) and hexaploid tritordeum accession HT22 (2n = 6x = 42; AABBH^ch^H^ch^) exhibiting the non-free threshing phenotype derived from the cross between H1 and T22. In addition, two free-threshing lines of hexaploid tritordeum obtained by chromosome substitution [36] were used: HT374 (5D/5H^ch^) and HT382 (1D/1H^ch^, 2D/2H^ch^). Plants were grown in a greenhouse with supplementary lights providing a day/night regime of 12/12 h at 22/16°C.

### RNA isolation

Tissues for RNA extractions were collected, immediately frozen by immersion in liquid nitrogen and stored at -80°C. RNA was isolated using TRIzol reagent (Invitrogen, Carlsbad, CA) according to the manufacturer's instructions, and treated with TURBO DNase (RNase-Free; Ambion, Warrington, UK) to eliminate any DNA contamination. The resulting RNA was stored at -80°C.

### Rapid amplification of 5' and 3' cDNA ends (5' and 3' RACE PCR)

The SMART RACE cDNA Amplification Kit (Clontech, Palo Alto, CA) was used for both 5'- and 3'- rapid amplification of cDNA ends. Four nested specific primers, two forward and two reverse (Table 3), were designed using the *H. vulgare *cv. Forester AP2-like protein mRNA sequence [GenBank: AY069953] as template and were used in combination with the primers provided by the kit. The RACE PCR products were cloned into pGEMT Easy vector (Promega, Madison, WI) and introduced into *Escherichia coli *(DH5α) competent cells by transformation. The resulting plasmid was isolated, purified using the Perfectprep Plasmid Mini Kit (Eppendorf, Hamburg, Germany), and sequenced.

### Full-length open reading frame isolation

The 3' RACE PCR sequences were used in combination with the *AP2 *sequence from *H. vulgare *cv. Forester [GenBank: AY069953] to design the primers AP2F4 and AP2newR (Table 3) to amplify the full open reading frame (ORF) of *AP2*-like gene from *H. chilense *lines H11, H208 and *H. vulgare *line H106. cDNA was synthesized using total RNA isolated from immature spikes of above lines using a Moloney Murine Leukemia Virus Reverse Transcriptase (M-MLV RT) (Invitrogen. Carlsbad, CA) in combination with oligo (dT)_12–18 _and random nonamers (Amersham Biosciences, Amersham, UK) according to the manufacturer's instructions. Full ORF was amplified by PCR using the following conditions: cDNA obtained from 50 ng of total RNA, 2 mM MgCl_2_, 0.2 mM dNTP, 0.2 μM of each primer, 1× CERTAMP buffer and 1 unit of CERTAMP Enzyme Mix (BIOTOOLS, Madrid, Spain) in a final volume of 25 μl. Cycling conditions were: 94°C 3 min, 35 cycles of 94°C 30 sec, 65°C 1 min and 72°C 2 min; and a final extension cycle of 72°C 7 min. PCR product was purified using the GFX PCR DNA Purification Kit (Amersham Biosciences, Amersham, UK) and sequenced.

### Edge-to-edge genomic DNA isolation

Genomic DNA was isolated from leaves using the DNAzol reagent (Invitrogen. Carlsbad, CA) according to the manufacturers' instructions. Primers used for amplification were AP2F4 and AP2newR (Table 3). PCR conditions for edge-to-edge genomic amplification were 50 ng of genomic DNA, 2 mM MgCl_2_, 0.2 mM dNTP, 0.2 μM of each primer, 1× CERTAMP buffer and 1 unit of CERTAMP Enzyme Mix (BIOTOOLS, Madrid, Spain) in a final volume of 25 μl. Cycling conditions were the following: 94°C 3 min, 35 cycles of 94°C 30 sec, 65°C 1 min and 72°C 4 min; and a final extension cycle of 72°C 7 min. PCR products were run in a 1% agarose gel and gel-purified using the QUIAquick Gel Extraction Kit (QUIAGEN Inc., Valencia, CA). Fragments were cloned into pGEMT Easy vector (Promega, Madison, WI) and introduced into *E. coli *(DH5α) competent cells by transformation. The resulting plasmid was isolated, purified using the Perfectprep Plasmid Mini Kit (Eppendorf, Hamburg, Germany), and sequenced. Three internal primers (F3APT2, F4APT2 and R5APT2) were designed and, in combination with the AP2F4 and AP2newR, used for the complete sequencing (Table 3).

### Quantitative real time PCR (qRT-PCR)

**Figure 7:**
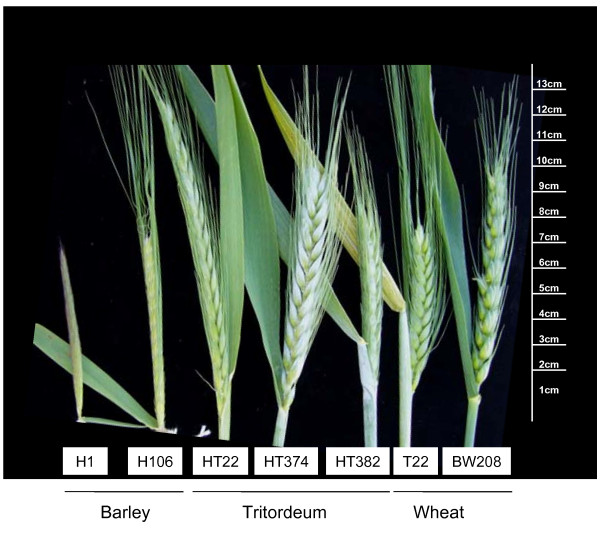
**Fully emerged spikes of *H. chilense *(H1), *H. vulgare *cv Betzes (H106), tritordeum (HT22, HT374 and HT382), *T. durum *(T22) and *T. aestivum *cv Bobwhite (BW208)**. The scale in cm is represented on the right.

Total RNA was isolated, and cDNA synthesised as described above, from the following tissues of *H. chilense *(H1), *H. vulgare *cv Betzes (H106), *T. durum *(T22), *T. aestivum *cv Bobwhite and tritordeum (lines HT22, HT374 and HT382): i) roots from 1 month old plants; ii) stems; iii) the fourth leaf from 1 month old plants; iv) non emerged spikes, and v) fully emerged spikes (Feeks scale 10.5). Non emerged spikes were classified as percentage of the fully emerged spike length (Figure 7).

qRT-PCR was carried out using the SYBR green dye marker on the ABI Prism 7000 (Applied Biosystems, Warrington, UK). qRT-PCR conditions were: cDNA obtained from 40 ng of total RNA, 0.4 μM of each primer and 1× SYBR Green Master Mix in a final volume of 25 μl. Two sets of primers were used for the specific amplification of part of the *AP2*-like gene from wheat (AP2*F2/AP2Ta*R2) and cultivated and wild barley (AP2*F2/AP2Hch*R2). The wheat *actin *mRNA [GenBank: AB181991] was used as reference gene using primers ActinF4 and ActinR4 (Table 3). Three replications of each reaction were performed with three biological samples for each tissue sample. Cycling conditions for qRT-PCR were the following: 95°C for 10 min, 40 cycles of 95°C for 30 sec and 60°C for 1 min. Primers efficiency was determined for all the genotypes. The mean and standard error for all replications were calculated for each point. Quantitative data generated were firstly analyzed using ABI Prism 7000 software following the steps described by Nolan *et al*. [37]. Expression data were then standardized using the Microsoft Excel Qgene template as described in Muller *et al*. [38].

### Bioinformatic analyses

Primer design was made with the Primer3Plus on-line application [39]. Other analyses and bioinformatics designs were performed with the Vector NTI 9.1.0 suite (Invitrogen, Carlsbad, CA). DNA and peptide-sequence similarities were determined by searches through the GenBank database, using the program BLAST (basic local alignment search tool) [40] on the website [41].

## Authors' contributions

JGH designed the primers and cloned the AP2-like genes. FP analysed the sequences. AM provided plant materials. FB designed the experiments. JGH, FP, AM and FB drafted the manuscript. All authors read and approved the final manuscript.

## Supplementary Material

Additional file 1 **Phylogenetic tree of the AP2-like proteins**. Phylogenetic tree based on the alignment of the AP2-like proteins obtained from the BLASTp of the *H. chilense *(H11 and H208) and *H. vulgare *cv. Betzes (H106) AP2 predicted proteins. The GenBank numbers of the AP2-like proteins are as following: *H. vulgare *[GenBank: AAL50205], *T. aestivum *[GenBank: AAU94922], *Z. mays *[GenBank: AAC05206], *O. sativa *[GenBank: AAO65862] and *A. thaliana *[GenBank: AAC13770]. The phylogenetic tree was calculated by the neighbour-joining method. Click here for file
