# Supplementary figures and images for: Comparative genomic analysis and expression of the APETALA2-like genes from barley, wheat, and barley-wheat amphiploids
Source: BMC Plant Biol. 2009 May 29;9:66. doi: 10.1186/1471-2229-9-66 (PMC2700811; doi:10.1186/1471-2229-9-66)

## Slide 1
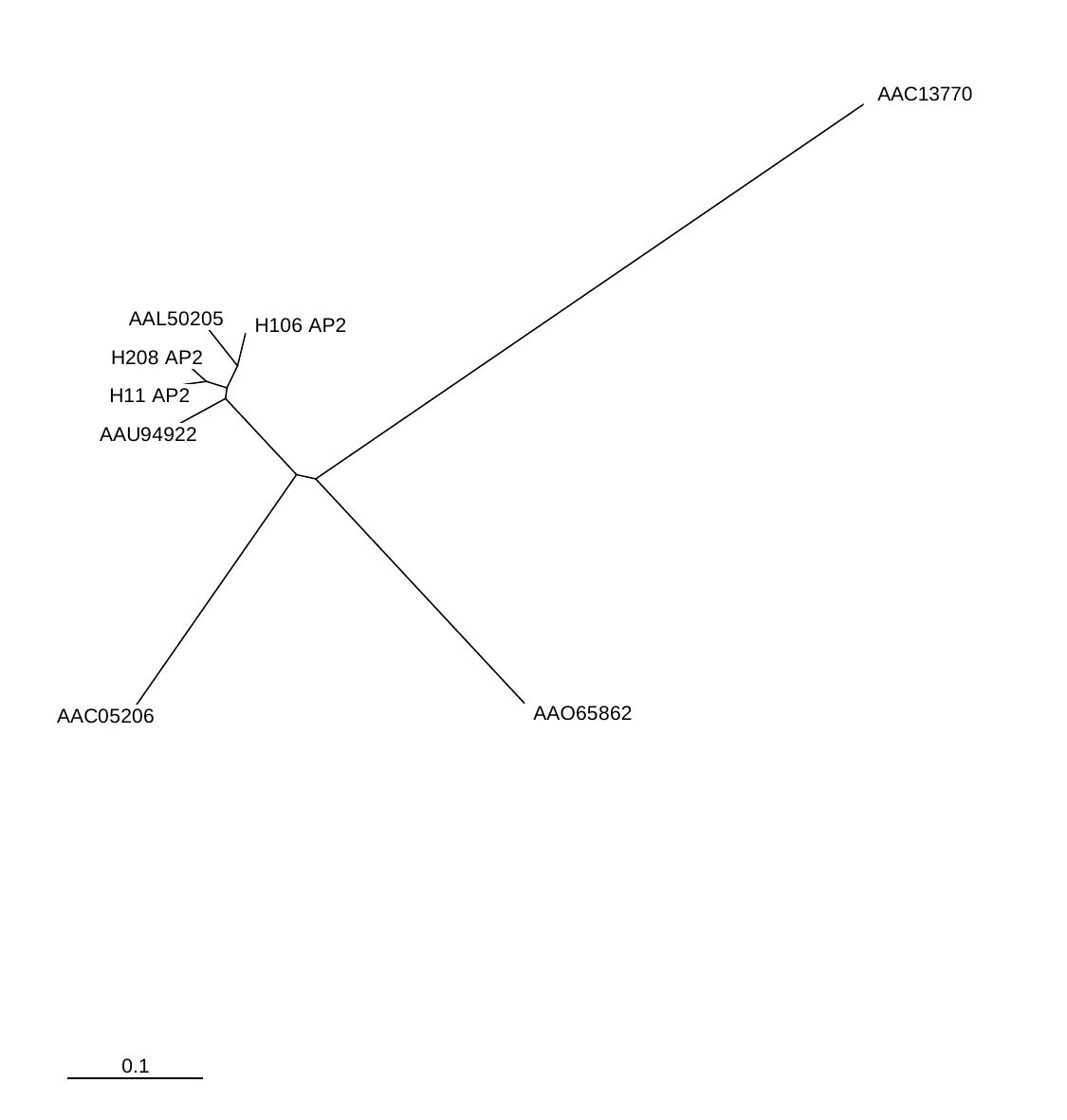

AAC13770

Supplement: Additional file 1 — Phylogenetic tree of the AP2-like proteins. Phylogenetic tree based on the alignment of the AP2-like proteins obtained from the BLASTp of the H. chilense (H11 and H208) and H. vulgare cv. Betzes (H106) AP2 predicted proteins. The GenBank numbers of the AP2-like proteins are as following: H. vulgare [GenBank: AAL50205], T. aestivum [GenBank: AAU94922], Z. mays [GenBank: AAC05206], O. sativa [GenBank: AAO65862] and A. thaliana [GenBank: AAC13770]. The phylogenetic tree was calculated by the neighbour-joining method. [file 1471-2229-9-66-S1.ppt]
